# Outcomes of trastuzumab therapy in HER2-positive early breast cancer patients: extended follow-up of JBCRG-cohort study 01

**DOI:** 10.1007/s12282-020-01057-4

**Published:** 2020-02-14

**Authors:** Hiroyasu Yamashiro, Hiroji Iwata, Norikazu Masuda, Naohito Yamamoto, Reiki Nishimura, Shoichiro Ohtani, Nobuaki Sato, Masato Takahashi, Takako Kamio, Kosuke Yamazaki, Tsuyoshi Saito, Makoto Kato, Tecchuu Lee, Katsumasa Kuroi, Toshimi Takano, Shinji Yasuno, Satoshi Morita, Shinji Ohno, Masakazu Toi, the JBCRG-C01 Collaborative Group, K. Yamagami, T. Morimoto, Y. Hasegawa, H. Shigematsu, M. Hosoda, H. Abe, D. Yotsumoto, H. Tanino, Y. Yamamoto, K. Hisamatsu, T. Aihara, H. Bando, H. Yoshibayashi, N. Tagaya, H. Doihara, K. Narui, H. Mukai, K. Aogi, S. Tsuyuki, Y. Kawabuchi, Y. Wada, Y. Kakugawa, Y. Moriguchi, Y. Kawaguchi, H. Suwa, F. Tanaka, H. Nakagomi, T. Ito, S. Nakamura, H. Takeuchi, M. Inokuchi, Y. Teramura, K. Ito, S. Sato, F. Yotsumoto, T. Okino, Y. Mitsudo, K. Yoshidome, Y. Tokunaga

**Affiliations:** 1 Department of Breast Surgery, Tenri Hospital, 200 Mishima-cho, Tenri, Nara 632-8552 Japan; 2 Department of Breast Oncology, Aichi Cancer Center Hospital, 1-1 Kanokoden, Chikusa-ku, Nagoya, Aichi 464-8681 Japan; 3 Surgery, Breast Oncology, National Hospital Organization Osaka National Hospital, 2-1-14 Hohenzaka, Chuo-ku, Osaka, 540-0006 Japan; 4 Division of Breast Surgery, Chiba Cancer Center, 666-2 Nitona-cho, Chuo-ku, Chiba, 260-8717 Japan; 5 Department of Breast Oncology, Kumamoto Shinto General Hospital, 3-2-65 Oe, Chuo-ku, Kumamoto City, Kumamoto 862-8655 Japan; 6 Division of Breast Surgery, Hiroshima City Hiroshima Citizens Hospital, 7-33 Motomachi, Naka-ku, Hiroshima-shi, Hiroshima, 730-8518 Japan; 7 Department of Breast Oncology, Niigata Cancer Center Hospital, 2-15-3 Kawagishi town, Chuo-ku, Niigata, 951-8566 Japan; 8 Department of Breast Surgery, NHO Hokkaido Cancer Center, 4-2 Kikusui, Shiroishi-ku, Sapporo, Hokkaido 003-0804 Japan; 9 Department of Breast, Endocrine and Pediatric Surgery, Tokyo Women’s Medical University, 8-1 Kawadacho, Shinjuku-ku, Tokyo, 162-8666 Japan; 10 Japanese Red Cross Hokkaido College of Nursing, 664-1 Akebono-cho, KitamiKitami, Hokkaido 090-0011 Japan; 11 Department of Breast Surgery, Japanese Red Cross Saitama Hospital, 1-5 Shintoshin, Chuo-ku, Saitama-shi, Saitama, 330-8553 Japan; 12 Kato Breast Surgery Clinic, 8-12 Nishiojicho, Kusatsu, Shiga 525-0037 Japan; 13 Department of Breast Surgery, Japanese Red Cross Kyoto Daiichi Hospital, 15-749 Honmachi, Kyoto Higashiyama-ku, Kyoto, 605-0981 Japan; 14 Department of Breast Surgery, Tokyo Metropolitan Cancer and Infectious Diseases Center Komagome Hospital, 3-18-22 Honkomagome, Bunkyo-ku, Tokyo, 113-8677 Japan; 15 Department of Medical Oncology, Toranomon Hospital, 2-2-2 Toranomon, Minato-ku, Tokyo, 105-8470 Japan; 16 Department of EBM Research, Institute for Advancement of Clinical and Translational Science, Kyoto University Hospital, 54 Kawaharacho, Shogoin, Sakyo-ku, Kyoto, 606-8507 Japan; 17 Department of Biomedical Statistics and Bioinformatics, Kyoto University Graduate School of Medicine, 54 Kawaharacho, Shogoin, Sakyo-ku, Kyoto, 606-8507 Japan; 18 Breast Oncology Center, The Cancer Institute Hospital of JFCR, 3-8-31, Ariake, Koto-ku, Tokyo, 135-8550 Japan; 19 Breast Cancer Unit, Kyoto University Hospital, 54 Kawaharacho, Shogoin, Sakyo-ku, Kyoto, 606-8507 Japan

**Keywords:** Breast cancer, HER2-positive breast cancer, Cohort study, Trastuzumab, Prediction model

## Abstract

**Background:**

Previous large trials of trastuzumab (TZM) demonstrated improved outcomes in patients with HER2-positive early breast cancer. However, its effectiveness and safety in Japanese patients is not yet clear. Recently, new anti-HER2 agents were developed to improve treatment outcomes, but the patient selection criteria remain controversial.

**Purpose:**

The aim of this study was to evaluate the long-term effectiveness of TZM therapy as perioperative therapy for HER2-positive operable breast cancer in daily clinical practice and to create a recurrence prediction model for therapeutic selection.

**Methods:**

An observational study was conducted in Japan (UMIN000002737) to observe the prognosis of women (*n* = 2024) with HER2-positive invasive breast cancer who received TZM for stage I–III C disease between July 2009 and June 2011. Moreover, a recurrence-predicting model was designed to evaluate the risk factors for recurrence.

**Results:**

The 5- and 10-year disease-free survival (DFS) rates were 88.9 (95% CI 87.5–90.3%) and 82.4% (95% CI 79.2–85.6%), respectively. The 5- and 10-year overall survival (OS) rates were 96% (95% CI 95.1–96.9%) and 92.7% (95% CI 91.1–94.3%), respectively. Multivariate analysis revealed that the risk factors for recurrence were an age of ≥ 70 years, T2 or larger tumors, clinically detected lymph node metastasis, histological tumor diameter of > 1 cm, histologically detected lymph node metastasis (≥ n2), and the implementation of preoperative treatment. The 5-year recurrence rate under the standard treatment was estimated to be > 10% in patients with a score of 3 or greater on the recurrence-predicting model.

**Conclusion:**

The recurrence-predicting model designed in this study may improve treatment selection of patients with stage I–III C disease. However, further studies are needed to validate the scores generated by this model.

**Electronic supplementary material:**

The online version of this article (10.1007/s12282-020-01057-4) contains supplementary material, which is available to authorized users.

## Introduction

Several randomized trials of trastuzumab (TZM) have demonstrated improved outcomes in patients with HER2-positive early breast cancer (EBC) [1–4]. We previously conducted the JBCRG C-01 cohort study, and reported the efficacy and safety of perioperative trastuzumab therapy for HER2-positive EBC [5]. However, the long-term outcomes are not yet clear. Recently, perioperative therapy for HER2-positive EBC has been improved via attempts to eliminate anthracycline [1, 6, 7], the development of anti-HER2 agents, such as neratinib [8] and pertuzumab [9], and shortening of the trastuzumab administration duration [10–12]. The current issue is to distinguish patients who require more potent treatment from those for whom administration must be de-escalated. Furthermore, due to the increased options for post-recurrence treatment [13, 14], there is a growing need to carry out surveillance for recurrence on an appropriate schedule. In this study, the effectiveness of TZM therapy as perioperative therapy for HER2-positive operable breast cancer in daily clinical practice was evaluated, and the clinical issues based on the updated data from the JBCRG C-01 study were examined.

## Patients and study design

An observational study was performed on patients aged over 20 years who were histologically diagnosed with invasive HER2-positive breast cancer stage I–III C and treated using TZM. Patients (*n* = 2024) from 56 institutions which participated in the Japan Breast Cancer Research Group (JBCRG) between July 2009 and June 2016 were registered in this study. All patients received perioperative TZM-containing therapy between January 2006 and June 2011 for at least 10 months. The data were finalized in August 2016. Forty-three patients who failed to meet the eligibility criteria were excluded and 1981 datasets were analyzed in this study.

The study protocol was approved by each institutional review board. We ensured that the subjects received a full explanation of the study according to the ethical guidelines for epidemiological studies and received their written informed consent or opt-out in accordance with the standards of the study centers. Data were managed by the Department of EBM Research, Institute for the Advancement of Clinical and Translational Science, Kyoto University Hospital, and the JBCRG Data Center. This study has been registered in the University Hospital Medical Information Network (UMIN), number UMIN000002737.

### Primary and secondary endpoints

The endpoints in this study were previously described [5]. In brief, the primary and secondary endpoints were disease-free survival (DFS) and overall survival (OS), respectively.

### Statistics

The Kaplan–Meier method was used to estimate DFS and OS curves. The Chi-squared (*x*^2^) test or Wilcoxon tests for categorical data and log-rank test for time-to-event endpoints provided two-sided *p* values, and a *p* value < 0.05 was considered significant. Cox regression analysis was used to estimate hazard ratios (HRs) and 95% confidence intervals (CIs). Covariates used in the multivariate model were age, clinical tumor stage, clinical nodal stage, estrogen receptor (ER)/progesterone receptor (PgR) status, HER2 status, histological/nuclear grade, menopausal status, and past medical history. Statistical analyses were performed using SAS ver. 9.2 (SAS Institute).

## Results

### Patient characteristics and treatment

The median follow-up period was 80.9 (5.0–132.2, mean 80.2) months. Baseline characteristics and treatments are summarized in Table 1. The expression of ER, PgR, and HER2, and grade were determined using biopsy specimens before preoperative treatment from patients who received preoperative therapy or surgical specimens from patients without preoperative therapy.

**Table 1:** Patient characteristics and treatment

	Number	(%)
Total	1981	100.0
Age		
< 35	70	3.5
35–49	554	28.0
50–59	713	36.0
60–69	501	25.3
≥ 70	143	7.2
Mean, SD	54.3	10.7
Max, Min	18	86
Menopausal state		
Premenopause	698	35.2
Postmenopause	1283	64.8
Tumor stage at initial diagnosis		
TX	5	0.3
Tis	27	1.4
T0	8	0.4
T1	637	32.2
T2	1030	52.0
T3	172	8.7
T4	102	5.1
Nodal status at initial diagnosis		
NX	4	0.2
N0	1131	57.1
N1	673	34.0
N2	130	6.6
N3	42	2.1
NA	1	0.1
Tumor grade		
1	179	9.8
2	633	34.5
3	1021	55.7
NA	148	7.4
ER/PgR status		
ER and/or PgR positive	895	45.9
ER and PgR negative	1057	54.1
NA	29	1.5
HER2 status		
IHC 3	1617	84.0
IHC ≤ 2 FISH+	264	13.7
FISH +	43	2.2
Not classified above	57	2.9
Neoadjuvant therapy performed	702	35.4
Adjuvant therapy performed	1974	99.6
Trastuzumab administration		
Preoperative only	26	1.3
Pre- and postoperative	440	22.2
Postoperative only	1515	76.5
Hormonal therapy performed	954	48.2
Surgery	954	48.2
Partial mastectomy	1021	51.6
Mastectomy	959	48.4
NA	1	0.1
Radiotherapy performed	1139	57.5

*LN* lymph node, *ER* estrogen receptor, *PgR* progesterone receptor, *IHC* immunohistochemical staining, *FISH* fluorescence in situ hybridization, *NA* not available

Mastectomy was performed for 959 patients and breast-conserving surgery for 1021 patients. A total of 1139 patients received postoperative radiotherapy and 954 patients received postoperative hormonal therapy.

In this observational study assessing the common use of TZM, we did not predetermine the treatment protocol. In brief, of the 702 patients who received preoperative therapy, 415 received TZM preoperatively and 26 received only TZM preoperatively. A total of 1279 patients received postoperative systemic therapy only, and 137 patients received TZM monotherapy (Table 2).

**Table 2:**
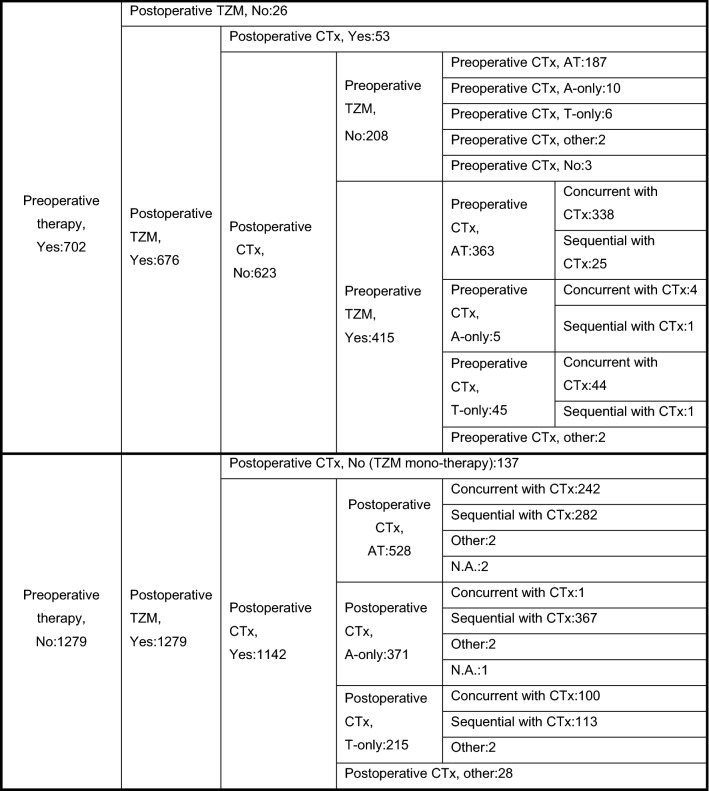
Systemic treatment (in detail)

*CTx* chemotherapy, *A* anthracycline, *T* taxane

### DFS and OS

**Fig. 1:**
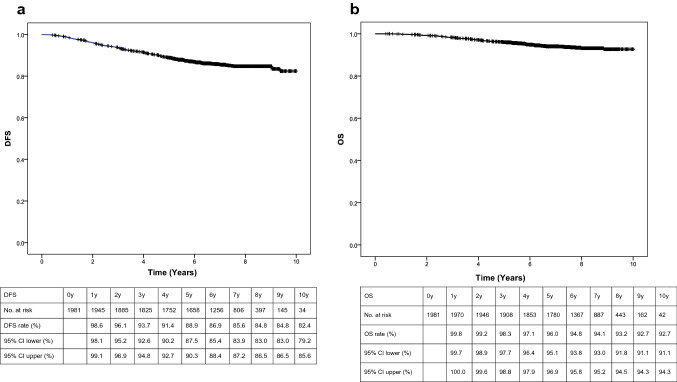
**a** Disease-free survival (DFS) for all patients. **b** Overall survival (OS) for all patients

The 5- and 10-year DFS rates were 88.9 (95% CI 87.5–90.3%) and 82.4% (95% CI 79.2–85.6%), respectively. The 5- and 10-year OS rates were 96% (95% CI 95.1–96.9%) and 92.7% (95% CI 91.1–94.3%), respectively (Fig. 1a, b).

### Timing of recurrence with respect to organs

**Fig. 2:**
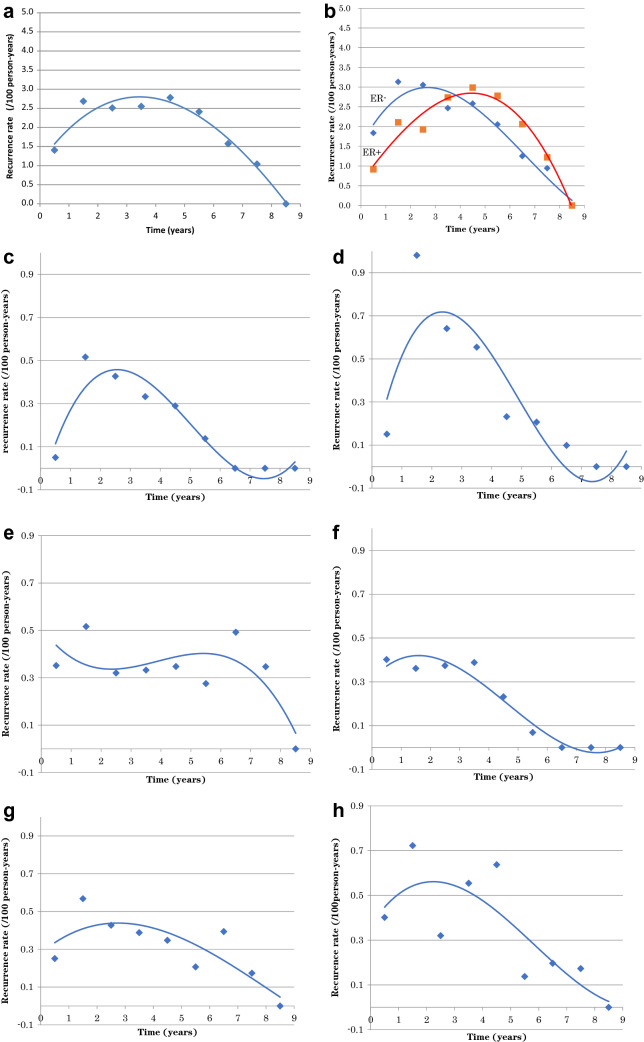
Annual hazard of recurrence. **a** All. **b** Stratified by ER status. **c**–**h** Stratified by first recurrence site, **c** liver, **d** lung, **e** chest wall, supraclavicular lymph node, and para sternal lymph node, **f** brain, **g** bone, **h** ipsilateral breast and axillary lymph node

We calculated the annual risk of first recurrence with respect to ER expression and sites (Fig. 2a–f). ER-negative disease often recurred earlier than ER-positive disease. With respect to the site of the first recurrence, the incidence of liver and lung metastasis reached a peak at 2–3 years after surgery. However, there was no peak in thoracic wall, supraclavicular, or parasternal lymph node recurrence after surgery, and a relatively high recurrence rate was continuously observed in a relatively late phase (5–7 years). The peak of brain metastasis was 1 year earlier than that of liver metastasis.

### Analysis of factors influencing DFS and a recurrence model

Univariate analyses comparing subgroups were performed using the log**-**rank test; hazard ratios (HRs) with 95% CIs were derived from Cox proportional hazards models (Table 3).

**Table 3:** Univariate analysis

	Univariate analysis				
	B	SE	HR (95% CI upper, lower)		*p* value
Age					
Per year, continuous	0.012	0.006	1.012	1.000, 1.023	0.044
< 60 vs. ≥ 60	0.317	0.124	1.373	1.077, 1.750	0.011
< 70 vs. ≥ 70	0.745	0.183	2.107	1.471, 3.019	< 0.001
Menopausal status					
Pre- vs. post-menopause	0.041	0.127	1.042	0.812, 1.337	0.746
T stage					
T1 vs. T2-4	0.950	0.162	2.586	1.884, 3.552	< 0.001
N sage					
N0 vs. N1-3	0.893	0.125	2.444	1.912, 3.123	< 0.001
Pathological tumor size					
≤ 1 cm vs. > 1 cm	0.383	0.142	1.467	1.110, 1.938	0.007
Pathological lymph node metastasis					
0 vs. 1–3 lymph nodes involved	0.429	0.145	1.536	1.156, 2.043	0.003
0 vs. ≥ 4 lymph nodes involved	1.152	0.149	3.164	2.365, 4.233	< 0.001
pN0 vs. pN +	0.715	0.121	2.044	1.611, 2.594	< 0.001
Grade					
Grade 1 vs. 2	− 0.085	0.224	0.918	0.592, 1.424	0.703
Grade 1 vs. 3	− 0.036	0.213	0.964	0.635	0.865
ER status					
Negative vs. positive	− 0.104	0.123	0.901	0.708, 1.146	0.396
HER2 status					
IHC 3 + vs. 2 + FISH +	− 0.023	0.180	0.977	0.687, 1.389	0.896
IHC 3 + vs. FISH +	0.321	0.360	1.378	0.681, 2.790	0.373
Surgery (type)					
Partial vs. total mastectomy	0.486	0.123	1.626	1.278, 2.070	< 0.001
Preoperative systemic therapy					
No vs. Yes	0.409	0.122	1.505	1.185, 1.912	0.001
CTx concurrent with TZM vs. sequential	0.196	0.425	1.217	0.529, 2.802	0.644
CTx concurrent with TZM vs. without CTx	− 9.998	230.841	0.000	0.000, inf	0.965
Postoperative systemic therapy					
No vs. yes	− 1.956	0.504	0.141	0.053, 0.380	< 0.001
CTx concurrent with TZM vs. sequential	− 0.006	0.182	0.994	0.696, 1.419	0.974
CTx concurrent with TZM vs. without CTx	0.472	0.176	1.604	1.136, 2.263	0.007
Chemotherapy					
No vs. yes (pre and/or postoperative)	− 0.380	0.206	0.684	0.457, .025	0.066

*B* regression coefficient, *SE* regression coefficient of regression coefficient, *HR* hazard ratio; *95% CI* 95% confidence interval

**Fig. 3:**
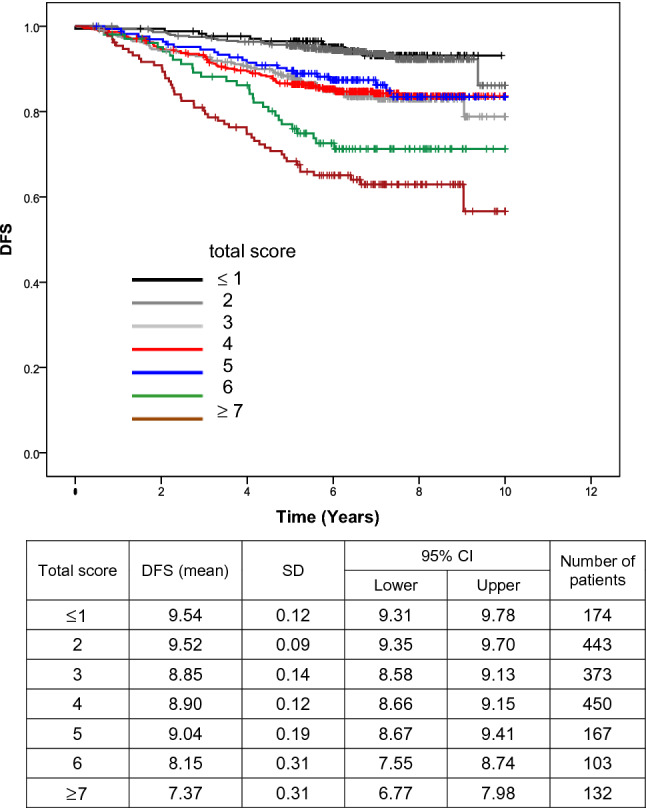
Estimated DFS of each score

Multivariate analysis revealed the risk factors for recurrence to be an age of ≥ 70 years, T2 or larger tumors, clinically detected lymph node metastasis, histological tumor diameter of > 1 cm, histologically detected lymph node metastasis(≥ n2), and the implementation of preoperative treatment. We prepared the risk score of recurrence based on the results of the multivariate analysis. When comparing the compulsive insertion method with the variable-increasing method using likelihoods, significant factors remained. Based on the coefficient calculated, the score ratio was calculated and the final score was determined by rounding off the values (Table 4). We calculated the 5-year recurrence risk for each total point (Table 5) and estimated the Kaplan–Meier curve for the DFS of each score (Fig. 3). The C-index was 0.653.

**Table 4:** Multivariate analysis and risk score

	Multivariate Model 1: Compulsive insertion method						Multivariate Model 2: Variable-increasing method using likelihoods						Score ratio	Score (point)
	B	SE	HR	HR (95% CI upper lower)		*p* value	B	SE	HR	HR (95% CI upper lower)		*p* value		
Age														
< 70	Ref						Ref							
≥ 70	0.850	0.191	2.340	1.610	3.400	< 0.001	0.850	0.191	2.340	1.610	3.400	< 0.001	2.193	2
Tumor stage														
T1	Ref						Ref							
T2–4	0.584	0.175	1.793	1.273	2.526	0.001	0.584	0.175	1.793	1.273	2.526	0.001	1.507	2
TX Tis T0	− 0.086	0.726	0.917	0.221	3.804	0.905	− 0.086	0.726	0.917	0.221	3.804	0.905		
Nodal status														
N0	Ref						Ref							
N1–3	0.388	0.162	1.473	1.073	2.023	0.016	0.388	0.162	1.473	1.073	2.023	0.016	1.000	1
NX	1.053	1.013	2.866	0.393	20.883	0.299	1.053	1.013	2.866	0.393	20.883	0.299		
Tumor size (pathological)														
< 1 cm	Ref						Ref							
≥ 1 cm	0.485	0.169	1.623	1.166	2.260	0.004	0.485	0.169	1.623	1.166	2.260	0.004	1.250	1
Lymph node metastasis														
n0	Ref						Ref							
n1	0.224	0.172	1.251	0.894	1.752	0.192	0.224	0.172	1.251	0.894	1.752	0.192		
n2–3	0.780	0.188	2.182	1.511	3.151	< 0.001	0.780	0.188	2.182	1.511	3.151	< 0.001	2.013	2
Preoperative systemic therapy														
No	Ref						Ref							
Yes	0.570	0.158	1.769	1.297	2.412	< 0.001	0.570	0.158	1.769	1.297	2.412	< 0.001	1.472	1

*B* regression coefficient, *SE* regression coefficient of regression coefficient, *HR* hazard ratio, *95% CI* 95% confidence interval, *ref* reference

**Table 5:** Risk score and estimated 5-year recurrence risk for each total point

Variable	Risk point
Age ≥ 70	2
T stage T2–4	2
N stage N1–3	1
Tumor size (pathological) ≥ 1 cm	1
Lymph node metastasis (≥ n2)	2
Preoperative systemic therapy (yes)	1

Total score	Probability (%)
0	3.5
1	4.7
2	6.4
3	8.6
4	11.6
5	15.6
6	21.1
7	28.4
8	38.4
9	51.8

## Discussion

In Japan, perioperative treatment using trastuzumab has markedly improved the outcomes of HER2-positive breast cancer patients. Even with perioperative chemotherapy using anthracycline or taxanes, approximately 25% of patients with HER2-positive breast cancer developed recurrence before trastuzumab became commercially available [15]. These results are consistent with those in the placebo group in a phase III clinical study of perioperative pertuzumab therapy (APHINITY study) [9]. No new AEs related to TZM were detected in this study.

The present study had several limitations. This cohort study was a single-arm observational study of TZM with or without chemotherapy in daily practice; therefore, the treatment effectiveness and clinicopathological features, including HR and HER2 status, were assessed by physicians. Treatment selection by each physician, including the surgical procedure, radiotherapy, and chemotherapy, may have affected the outcomes. The need for chemotherapy, especially for patients over 70 years of age, was not significant in this study, but conclusions should be carefully held until results of a randomized study are available.

An age of ≥ 70 years, grade of T2 or higher, clinically detected lymph node metastasis, histological tumor diameter of ≥ 1 cm, and histologically detected lymph node metastasis were extracted as prognostic factors, similar to the previous report [5]. Subgroup analysis in the APHINITY trial [9] also demonstrated the additive effects of pertuzumab in patients with lymph node metastasis or elderly patients. In this analysis, our recurrence-predicting model included the clinical N stage and pathological node-positive status. When employing the variable-increasing method with the likelihood ratio as mutually independent factors on multivariate analysis, these two factors remained.

Furthermore, the significance of histological node-positive status (*n*) and pathological tumor size (*t*) were suggested to depend on the presence of preoperative treatment; therefore, an additional subgroup analysis regarding the presence of preoperative treatment was performed. There was no preoperative treatment-related difference in the values on DFS of *n* and *t* (Supplement 1). Furthermore, we directly investigated the interaction between the presence of preoperative treatment and lymph node metastasis or pathological tumor size by analysis involving an interaction item, but it was insignificant. The influence of lymph node metastasis or pathological tumor size on DFS may be similar regardless of the presence of preoperative treatment. Indeed, even if preoperative chemotherapy results in pn0, the risk of recurrence was higher than if it was N0 before the start of treatment (Supplement 2).

In the guidelines, regular imaging, such as CT, is not recommended for asymptomatic patients [16]. In our study, the timing and annual risk of recurrent HER2-positive breast cancer were characterized by each organ. In particular, the start of treatment for recurrence in the phase of restricted tumor burden in patients with brain or bone metastases may minimize complications or treatment-related adverse effects. As the number of treatment options, such as pertuzumab [13] and T-DM1 [14], for metastatic or recurrent HER2-positive breast cancer has recently increased, this should be reflected in follow-up plans [17].

Recently, perioperative therapy for HER2-positive EBC has been improved via attempts to eliminate anthracycline [1, 6, 7], the development of anti-HER2 drugs with different actions, such as neratinib [8] and pertuzumab [9], and shortening of the trastuzumab administration period [10, 11]. If the risk of recurrence is high, the addition of pertuzumab or extension of neratinib treatment should be considered. Patients who fail to achieve pathological CR following neoadjuvant HER2-targeted therapy (along with chemotherapy) are also at increased risk for recurrence. For such patients, T-DM1 should be considered [18]. On the other hand, if the risk is low, treatment using short-term trastuzumab therapy may be successful. To optimize such treatment, it is necessary to comprehensively understand the risk of recurrence. Although the current staging is based on clinicopathological characteristics, our findings suggest that host factors, such as age, are included. Our recurrence model may be useful for future studies after validation.

## Conclusion

As this recurrence model was created based on the data from an observational study, validation is necessary. However, it may facilitate calculation of the risk of recurrence, thereby improving treatment selection.

## Electronic supplementary material

Below is the link to the electronic supplementary material.

Supplementary file1 (DOCX 72 kb)

## References

[1] Slamon D, Eiermann W, Robert N (2011). Adjuvant trastuzumab in HER2-positive breast cancer. N Engl J Med..

[2] Perez EA, Romond EH, Suman VJ (2011). Four-year follow-up of trastuzumab plus adjuvant chemotherapy for operable human epidermal growth factor receptor 2-positive breast cancer: joint analysis of data from NCCTG N9831 and NSABP B-31. J Clin Oncol..

[3] Spielmann M, Roche H, Delozier T (2009). Trastuzumab for patients with axillary-node-positive breast cancer: results of the FNCLCC-PACS 04 trial. J Clin Oncol..

[4] Gianni L, Dafni U, Gelber RD (2011). Treatment with trastuzumab for 1 year after adjuvant chemotherapy in patients with HER2-positive early breast cancer: a 4-year follow-up of a randomised controlled trial. Lancet Oncol..

[5] Yamshiro H, Iwata H, Masuda N (2015). Outcomes of trastuzumab therapy in HER2-positive early breast cancer patients. Int J Clin Oncol..

[6] Tolaney SM, Barry WT, Dang CT (2015). Adjuvant paclitaxel and trastuzumab for node-negative, HER2-positive breast cancer. N Engl J Med..

[7] Jones SE, Collea R, Paul D (2013). Adjuvant docetaxel and cyclophosphamide plus trastuzumab in patients with HER2-amplified early stage breast cancer: a single-group, open-label, phase 2 study. Lancet Oncol..

[8] Martin M, Holmes FA, Ejlertsen B (2017). Neratinib after trastuzumab-based adjuvant therapy in HER2-positive breast cancer (ExteNET): 5-year analysis of a randomised, double-blind, placebo-controlled, phase 3 trial. Lancet Oncol..

[9] von Minckwitz G, Procter M, de Azambuja E (2017). Adjuvant Pertuzumab and Trastuzumab in early HER2-positive breast cancer. N Engl J Med..

[10] Joensuu H, Bono P, Kataja V (2009). Fluorouracil, epirubicin, and cyclophosphamide with either docetaxel or vinorelbine, with or without trastuzumab, as adjuvant treatments of breast cancer: final results of the FinHer Trial. J Clin Oncol..

[11] Pivot X, Romieu G, Debled M (2013). 6 months versus 12 months of adjuvant trastuzumab for patients with HER2-positive early breast cancer (PHARE): a randomised phase 3 trial. Lancet Oncol..

[12] Earl HM, Hiller L, Vallier AL (2019). 6 versus 12 months of adjuvant trastuzumab for HER2-positive early breast cancer (PERSEPHONE): 4-year disease-free survival results of a randomised phase 3 non-inferiority trial. Lancet.

[13] Baselga J, Cortes J, Kim SB (2012). Pertuzumab plus trastuzumab plus docetaxel for metastatic breast cancer. N Engl J Med..

[14] Verma S, Miles D, Gianni L (2012). Trastuzumab emtansine for HER2-positive advanced breast cancer. N Engl J Med..

[15] Yamashiro H, Takada M, Nakatani E (2013). Prevalence and risk factors of bone metastasis and skeletal related events in patients with primary breast cancer in Japan. Int J Clin Oncol.

[16] Schnipper LE, Smith TJ, Raghavan D (2012). American Society of Clinical Oncology identifies five key opportunities to improve care and reduce costs: the top five list for oncology. J Clin Oncol..

[17] Runowicz CD, Leach CR, Henry NL (2016). American Cancer Society/American Society of Clinical Oncology Breast Cancer Survivorship Care Guideline. J Clin Oncol..

[18] Minckwitz G, Huang CS, Mano MS (2018). Trastuzumab emtansine for residual invasive HER2-positive breast cancer. N Engl J Med.

