# Supplementary material for: Outcomes of trastuzumab therapy in HER2-positive early breast cancer patients: extended follow-up of JBCRG-cohort study 01
Source: Breast Cancer. 2020 Feb 14;27(4):631–41. doi: 10.1007/s12282-020-01057-4 (PMC7297820; doi:10.1007/s12282-020-01057-4)
Supplement: Supplementary file 1 — Supplementary file1 (DOCX 72 kb) [file 12282_2020_1057_MOESM1_ESM.docx]

**Supplement 1**

Subgroup analysis regarding the presence of preoperative treatment on values as predictive indices N or n and T or t.

|  |  |  | **Univariate** | | | |  | **Multivariate** | | | |
| --- | --- | --- | --- | --- | --- | --- | --- | --- | --- | --- | --- |
|  |  |  | **HR** | **95%CI** | | **P-value** |  | **HR** | **95%CI** | | **P-value** |
| **Without preoperative systemic therapy** | | |  |  |  |  |  |  |  |  |  |
|  | N stage | |  |  |  |  |  |  |  |  |  |
|  |  | N0 vs. N1-3 | 2.356 | 1.719 | 3.229 | **<0.001** |  | 1.643 | 1.095 | 2.464 | **0.016** |
|  | Pathological lymph node metastasis | |  |  |  |  |  |  |  |  |  |
|  |  | pN0 vs. pN+ | 2.317 | 1.674 | 3.207 | **<0.001** |  | 1.722 | 1.133 | 2.617 | **0.011** |
| **With preoperative systemic therapy** | | |  |  |  |  |  |  |  |  |  |
|  | N stage | |  |  |  |  |  |  |  |  |  |
|  |  | N0 vs. N1-3 | 2.380 | 1.484 | 3.815 | **<0.001** |  | 1.906 | 1.155 | 3.144 | **0.012** |
|  | Pathological lymph node metastasis | |  |  |  |  |  |  |  |  |  |
|  |  | pN0 vs. pN+ | 2.256 | 1.562 | 3.258 | **<0.001** |  | 1.849 | 1.252 | 2.729 | **0.002** |
| **Without preoperative systemic therapy** | | |  |  |  |  |  |  |  |  |  |
|  | T stage | |  |  |  |  |  |  |  |  |  |
|  |  | T1 vs. T2-3 | 2.587 | 1.809 | 3.698 | **<0.001** |  | 2.348 | 1.630 | 3.382 | **<0.001** |
|  | Pathological tumor size | |  |  |  |  |  |  |  |  |  |
|  |  | <1 cm vs. ≥1 cm | 2.203 | 1.294 | 3.753 | **0.004** |  | 1.633 | 0.937 | 2.846 | 0.084 |
| **With preoperative systemic therapy** | | |  |  |  |  |  |  |  |  |  |
|  | T stage | |  |  |  |  |  |  |  |  |  |
|  |  | T1 vs. T2-3 | 1.871 | 0.764 | 4.584 | 0.170 |  | 1.848 | 0.753 | 4.534 | 0.180 |
|  | Pathological tumor size | |  |  |  |  |  |  |  |  |  |
|  |  | <1 cm vs. ≥1 cm | 2.083 | 1.430 | 3.036 | **<0.001** |  | 2.084 | 1.430 | 3.037 | **<0.001** |

**Supplement 2**

Kaplan-Meier curve stratified by change in nodal status after preoperative systemic therapy

N0 →ypn0

N1 →ypn0

N1 →ypn1-3

Log-rank test: p<0.001

**Time (Years)**
